# Development of a Web-Based Intervention for Middle Managers to Enhance Resilience at the Individual, Team, and Organizational Levels in Health Care Systems: Multiphase Study

**DOI:** 10.2196/67263

**Published:** 2025-02-05

**Authors:** Eva Gil-Hernández, Irene Carrillo, Jimmy Martin-Delgado, Daniel García-Torres, José Joaquín Mira

**Affiliations:** 1 ATENEA Research Group Foundation for the Promotion of Health and Biomedical Research of Valencia Region (FISABIO) Alicante Spain; 2 Health Psychology Department Universidad Miguel Hernández Elche Spain; 3 Junta de Beneficencia de Guayaquil Hospital de Especialidades Alfredo Paulson Portoviejo Ecuador; 4 Instituto de Investigación e Innovación en Salud Integral Facultad de Ciencias de la Salud Universidad Católica de Santiago de Guayaquil Guayaquil Ecuador; 5 Conselleria de Sanidad Health Centre Hospital Provincial-Pla Alicante-Sant Joan Health District Alicante Spain

**Keywords:** resilience, health care professionals, web-based intervention, middle management, well-being, patient safety

## Abstract

**Background:**

Health care institutions face high systemic risk due to the inherent uncertainty and complexity of their operations. This often leads to stressful incidents impacting the well-being of health care professionals, which can compromise the effectiveness of health care systems. Enhancing resilience among health care professionals is essential for maintaining high-quality care and ensuring patient safety. The role of middle managers is essential to ensure the response capacity of individuals and teams.

**Objective:**

This study aims to develop a web-based intervention aimed at middle management to enhance individual, team, and organizational resilience.

**Methods:**

An observational study was conducted in 3 phases: design, validation, and pilot study. The study was initiated in February 2022 and concluded in June 2023. Phase 1 involved designing the content for the web-based tool based on a comprehensive review of critical elements around resilience. Phase 2 included validation by an international panel of experts who reviewed the tool and rated it according to a structured grid. They were also encouraged to highlight strengths and areas for improvement. Phase 3 involved piloting the tool with health care professionals in Ecuador to refine the platform and assess its effectiveness. A total of 458 people were invited to participate through the Institutional Course on Continuous Improvement in Health Care Quality and Safety offered by the Ministry of Public Health of Ecuador.

**Results:**

The tool, eResiliencia, was structured into 2 main blocks: individual and team resilience and organizational resilience. It included videos, images, PDFs, and links to dynamic graphics and additional texts. Furthermore, 13 (65%) of the 20 experts validated the tool, rating content clarity at an average of 4.5 (SD 0.7) and utility at an average of 4.7 (SD 0.5) out of 5. The average overall satisfaction was 9.3 (SD 0.6) out of 10 points, and feedback on improvements was implemented. A total of 362 health care professionals began the intervention, of which 218 (60.2%) completed preintervention and postintervention questionnaires, with significant knowledge increases (*P*<.001). Of the 362 health care professionals, 146 (40.3%) completed the satisfaction questionnaire, where overall satisfaction was rated at an average of 9.4 (SD 1.1) out of 10 points.

**Conclusions:**

The eResiliencia web-based platform provides middle managers with resources to enhance resilience among their teams and their components, promoting better well-being and performance, even under highly stressful events. Future research should focus on long-term impacts and practical applications in diverse clinical settings.

## Introduction

### Systemic Risk

Health care institutions are considered high-reliability organizations due to the inherent systemic risk associated with the activities they conduct [1]. Uncertainty and complexity, inherent in health care delivery along with other factors, contribute to the occurrence of higher stressful incidents that sometimes result in harm (adverse events) [2]. Health care professionals are exposed almost daily to stressful situations that can deteriorate their occupational well-being, thus compromising the effectiveness of health care systems [3]. In addition, the impact and demands stemming from unexpected health outcomes and health care crises further exacerbate this situation. Ultimately, the post–COVID-19 pandemic period has underscored a reality that has always existed but has not been adequately addressed until now: the well-being of health care professionals is a necessary condition to ensure the effectiveness of health care systems and the quality of care [4].

### Professional Well-Being

In clinical contexts, situations with the potential to deteriorate the well-being of professionals are varied. These situations can range from those related to patient safety, such as near-misses, poor prognoses, unexpected health outcomes, a patient’s deteriorating condition, preventable and unavoidable adverse events, and the unexpected death of a patient, to those more associated with working conditions or structural factors. These include a lack of resources, high care pressure, elevated staff turnover, increased demand, violence against professionals, and moral injury. They can have an emotional impact on professionals, which affects their habits, practices, or clinical decisions [5]. When this behavior change occurs in the undesired direction, we may encounter defensive practices, burnout, or detachment that often entail assuming unnecessary risks for the patient and additional costs for the system [6,7].

### Vicious Cycle

Even in the absence of behavioral change, the loss of professional well-being is accompanied by an exponential increase in the risk of human error in patient care, leading to lower performance and a deterioration in the quality of care provided to patients [8]. This worsening of outcomes can, in turn, contribute to the occurrence of new errors and safety incidents. Thus, we find workers in a vicious cycle where the loss of well-being, the inability to regain balance and face stressful situations, and the quality of care are intimately related [5,9]. Hence, preserving and enhancing the resilience of professionals is a key element in ensuring the proper functioning of health care systems, the quality of care, patient safety, and achieving better health outcomes [10].

### Resilience

Resilience refers to the ability of individuals, teams, and organizations to adapt, recover, and grow in the face of stress, adversity, and challenging circumstances [11]. In the context of health care, it enables professionals to maintain their well-being and continue to provide high-quality care despite the inevitable challenges they encounter. This not only benefits the individuals themselves but also enhances the safety and effectiveness of care provided by health care teams [12].

### Individual Differences

In addition to the inherent risk in health care activities, other cultural aspects can also be a source of distress for health care professionals and may even constitute a barrier to the process of recovering well-being once it has been compromised and are also directly related to values. From an individual perspective, health care professionals share a set of ideals focused on doing good and prioritizing the well-being of others. While these ideals are desirable for good performance, their rigid application in critical situations can intensify distress or hinder constructive coping and professional recovery [13].

### Organizational Culture

At the organizational level, the institution’s culture plays a key role in determining how professionals feel, interpret, and cope with stressful situations. The prevailing professional culture in the health care sector is characterized by strong ideals of perfection that often lead to a negative and unconstructive view of human response, considering someone weak for asking for help or being unable to cope with a stressful situation. Understanding these causes allows for designing and implementing barriers that prevent or reduce the likelihood of succumbing to stressful situations occurring in the future.

### Challenge

The World Health Organization has incorporated resilience in health care systems into the Global Patient Safety Action Plan 2021-2030 through strategy 2.4: providing a strong human factor and ergonomics-based perspective and contributing to strengthening the resilience of health care organizations and clinical practices [14]. This initiative aims to address a basic need for the proper functioning of health care systems, which is to take care of those who care.

We intend to address this need not only from a reactive approach when things go wrong but also by creating resilient teams and providing middle managers with tools to reinforce the resilience of professionals, teams, and the health care organization to ensure their ability to respond to critical situations. We believe that middle managers, due to their strategic position in the institution’s structure, are key agents for the development of resilience at all levels; therefore, the materials of this project are specifically aimed at this group.

## Methods

### Study Design

**Figure 1:**
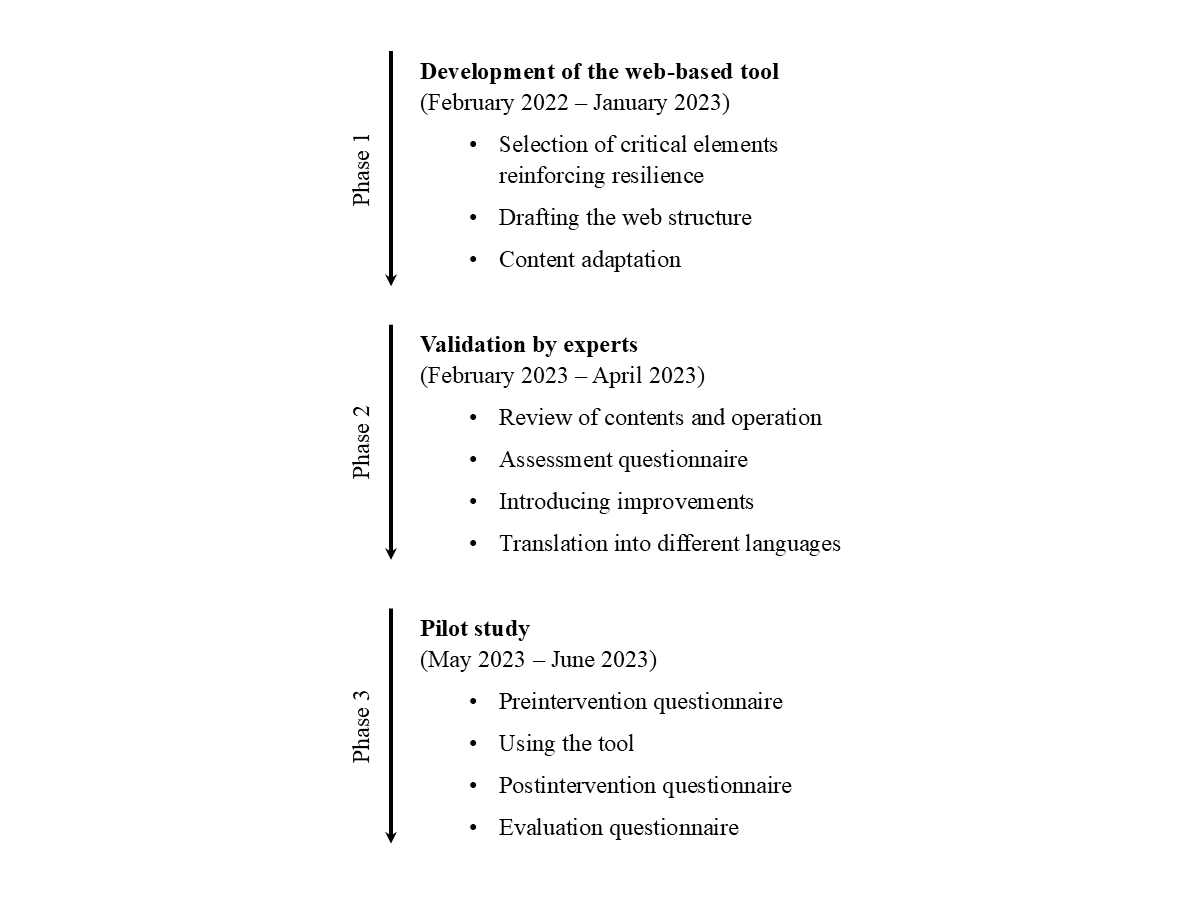
Flowchart of project phases and steps.

An observational study was developed in 3 phases: development of the web-based tool (phase 1), validation by experts (phase 2), and piloting with professionals (phase 3; Figure 1). Primary care and hospital were defined as usual settings for the new tool named eResiliencia. The established 8 principles for learning tools that aim to support the translation of resilience into practice by Haraldseid-Driftland et al [15] were used. In this study, resilience in health care was defined as the capacity to adapt to challenges and changes at different system levels to maintain high-quality care [12].

The study was developed by a core group involving a multidisciplinary team supported by a panel of international experts in health administration, occupational safety, and patient safety. It was initiated in February 2022 and concluded in June 2023.

### Phase 1: Development of the Web-Based Tool

To design the content for the web-based tool (phase 1), a comprehensive search of critical elements reinforcing resilience was conducted, culminating in a preliminary selection from among the most frequently cited elements. This included the revision of reviews and systematic reviews (after 2019) on the concept of resilience, experimental studies measuring the effectiveness of recent programs designed to enhance the resilience of health care professionals, and the content of different scales for measuring resilience. In particular, this search was aimed at identifying experiences and successful strategies [16].

**Figure 2:**
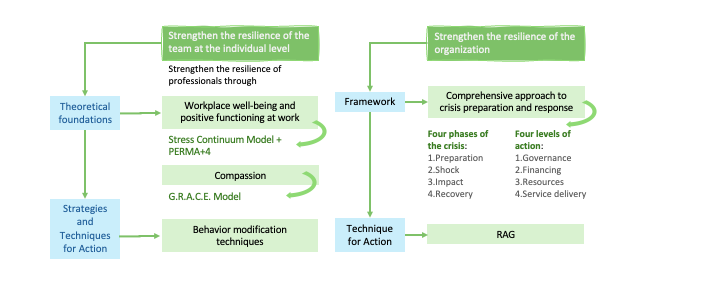
eResiliencia theoretical framework. PERMA+4: PERMA+4 Well-Being model; RAG: Resilience Analysis Grid.

The eResiliencia intervention is built on a robust theoretical framework that integrates principles of resilience, human behavior, and organizational adaptability to address the complex challenges faced by health care systems (Figure 2). This approach emphasizes strengthening resilience through the development of 5 essential traits: self-control, which involves regulating emotions and behavior under pressure; adaptability or the flexibility to adjust to changes and demands; optimism, characterized by a positive outlook and confidence in overcoming challenges; self-sufficiency, reflecting the ability to independently manage difficulties; and persistence, the sustained effort to persevere in the face of adversity.

The intervention draws from the 5-tier model proposed by Seys et al [17], which combines preventive and supportive measures to build resilience. Levels 1 and 2 focus on proactive strategies such as prevention and self-care, while levels 3 to 5 offer structured peer and clinical support to address resilience at both individual and organizational levels. This multilevel approach ensures that resilience-building efforts are comprehensive and adaptable to the diverse contexts within health care settings.

eResiliencia operates across 3 interconnected levels: individual, team, and organizational. It uses both proactive and reactive strategies to enhance resilience. Proactive measures aim to build capacity before crises occur, using tools such as stress management models and workplace well-being frameworks. Reactive strategies focus on recovery and learning following critical events, ensuring that individuals and organizations can adapt effectively to challenges.

The intervention is informed by several evidence-based models, including the stress continuum model [18], which helps monitor and manage stress; the PERMA+4 Well-Being model [19], which promotes workplace well-being through its multidimensional framework; the G.R.A.C.E. model [20], which supports health care workers in cultivating compassion and managing burnout; and the Resilience Analysis Grid [21], which was applied to assess and enhance team response capacity in unexpected events. In addition, eResiliencia incorporates a set of behavior change techniques [22] designed to equip middle managers with targeted strategies for promoting workplace well-being. These techniques are tailored to the unique cultural and organizational contexts of health care systems, ensuring their relevance and practicality.

Following this, a second step involved drafting the web structure to accommodate the intended contents effectively. Subsequently, a process of content adaptation to the health care context ensued, wherein various support materials for the tool were developed. These materials underwent 2 rounds of internal review before arriving at version 1.0, ensuring their alignment with the intended objectives. The first version of the web platform was developed in Spanish and hosted on the Miguel Hernández University of Elche (Spain) servers, which comply with security requirements and regulations. A total of 11 months were spent to complete this phase, commencing in February 2022.

### Phase 2: Validation by Experts

Version 1.0 underwent a review and validation process by an international panel of experts between February and April 2023. A total of 20 professionals integrated this panel, comprising 14 (70%) from Spain, 2 (10%) from Colombia, 1 (5%) from Argentina, 1 (5%) from Ecuador, 1 (5%) from Brazil, and 1 (5%) from Chile. The experts’ identification was made using the snowball approach. All experts were informed of the pursued objective and the approximate time commitment and were requested voluntary, unpaid participation. Of the 20 professionals, 11 (55%) were women and 9 (45%) were men, and all met the criterion of a minimum of 10 years of professional experience. It was ensured that no personal evaluation would be disclosed, and communications with them were conducted via telephone or email.

Following a comprehensive examination of all contents and their structural organization, experts engaged were required to respond to a rubric designed to evaluate various aspects. This rubric used a scale ranging from 1 to 5 to assess the clarity of the content and its utility and applicability, grading from completely disagree to completely agree on whether the materials facilitated learning strategies to foster the resilience of professionals or teams under their purview, their likelihood to recommend the tool to other colleagues, and their intention to apply learned tools or strategies. In addition, experts were encouraged to identify strengths and areas for improvement and to rate their overall satisfaction with the web-based intervention and its contents on a scale from 1 to 10, where the higher the score, the greater the satisfaction. The level of agreement among evaluators was assessed to inform decisions regarding aspects requiring changes. All feedback and suggested improvements were meticulously implemented to enhance the quality of the content. Subsequently, the refined version of the tool was translated into English, Portuguese, and Valencian. Careful attention was given to ensuring equivalence across all versions through a process of back translation. Necessary adjustments were made to ensure the adequacy and coherence of the system across languages. An adaptation was also made to the idioms and expressions commonly used in Latin American Spanish. This procedure also ensured that social and cultural aspects specific to each country were considered.

### Phase 3: Pilot Study

From May 1 to June 30, 2023, a pilot study was conducted in a real setting in Ecuador. The participants’ feedback served to refine the platform and the tool. A broader cohort of 458 health care professionals from Ecuador was invited to participate. They served as executives or managers in one of the country’s public health care networks. This invitation was extended through the Institutional Course on Continuous Improvement in Health Care Quality and Safety offered by the Ministry of Public Health of Ecuador with the support of the Miguel Hernández University, Spain. The invitation was formally extended to all professionals from the management teams of public health care institutions in the country, including physicians, nurses, and other professional profiles. They assessed whether they wished to participate in this training, ensuring their data and performance were completely anonymized. Participants were required to complete a preintervention questionnaire at the moment of registration to access the contents of the web-based tool. This questionnaire comprised 20 questions assessing the knowledge areas to be addressed. In Multimedia Appendix 1, the topics addressed by each question can be consulted. After 7 days, a postintervention questionnaire containing the same questions was made available to assess content assimilation. Participants had the option to retake this postintervention questionnaire up to a maximum of 10 times, with a minimum score of 8 out of 10 required to qualify for a proficiency certificate. Following the completion of the postintervention questionnaire, participants were presented with an evaluation questionnaire consisting of the same questions posed to the experts during phase 2.

### Data Analysis

In this study, both quantitative and qualitative analyses were conducted. The profile of participants who completed all phases of the iteration was compared with those who dropped out and did not finish to identify potential differences. For phase 2, descriptive analyses were performed on the rubric scores, and content analyses were conducted for open-ended responses. Fleiss κ was used to analyze the level of agreement among evaluators. The response options were recoded into 2 categories: “agree” (values 4 and 5) and “disagree” (values 1, 2, and 3), evaluating the overall agreement among experts across the 5 variables (clarity, utility, intention to recommend, intention to apply, and eResiliencia).

Similarly, in phase 3, the same analyses as in phase 2 were performed for the evaluation questionnaire. Descriptive statistics were conducted for both the preintervention and postintervention questionnaires. To assess differences between the means of such questionnaires, the Wilcoxon signed rank test was used. In addition, the McNemar statistic was calculated to compare the number of correct responses before and after the use of the web-based tool for each question. Qualitative analysis also considered the frequency of positive comments and improvement suggestions made by participants at the end of the structured survey. These comments, expressed in natural language, were categorized by 2 researchers (EG-H and IC) to facilitate their documentation. Finally, sociodemographic data were analyzed using descriptive statistics.

### Ethical Considerations

In accordance with the Ecuadorian Ministerial Agreement AM00005-2022 and the Spanish Law on Biomedical Research (14/2007), studies conducted through anonymous surveys that do not collect health-related data are exempt from ethical review. This study was fully compliant with all regulatory standards for personal data protection. Ethical principles for medical research involving human participants included in the guidelines set by the Helsinki Declaration were followed. Informed consent for study participation was obtained at the time of registration on the platform, whereby individuals were required to select the corresponding checkbox, with instructions provided regarding the process for revoking their participation. No form of financial compensation was provided for participation.

## Results

### Phase 1: Development of the Web-Based Tool

A total of 42 documents were deemed relevant and were analyzed in depth to extract lessons learned and proposals for the design of eResiliencia (Table 1).

**Table 1:** Documents considered when designing the content of eResiliencia.

Name		Study
**Resilience scales (n=7)**		
	Resilience at Work Scale	Winwood et al [23], 2013
	50-Item Resilience Questionnaire	The Psychometric Project [24], 2013
	Brief Resilience Scale	Smith et al [25], 2008
	Connor-Davidson Resilience Scale	Connor and Davidson [26], 2003
	Resilience Scale for Adults	Friborg et al [27], 2003
	Resilience Scale	Wagnild and Young [28], 1993
	Dispositional Resilience Scale	Bartone et al [29], 1989
**Resilience concept (n=11)**		
	Psychological Resilience: An Affect-Regulation Framework	Troy et al [30], 2023
	A Simultaneous Concept Analysis of Resilience, Coping, Posttraumatic Growth, and Thriving	Bowling et al [31], 2022
	An Approach to the Unified Conceptualization, Definition, and Characterization of Social Resilience	Moya and Goenechea [32], 2022
	Integrative Review of the Recent Literature on Human Resilience: From Concepts, Theories, and Discussions Towards a Complex Understanding	Métais et al [33], 2022
	Resilience: An Integrated Review	Daly [34], 2020
	Psychological Resilience as an Emergent Characteristic for Well-Being: A Pragmatic View	Tay and Lim [35], 2020
	What Does Resilience Signify? An Evaluation of Concepts and Directions for Future Research	Infurna [36], 2020
	Nurse Resilience: A Concept Analysis	Cooper et al [37], 2020
	What Are the Factors Affecting Resilience in Health Professionals? A Synthesis of Systematic Reviews	Huey and Palaganas [38], 2020
	Toward a Transversal Definition of Psychological Resilience: A Literature Review	Sisto et al [11], 2019
	Resilience as a Multimodal Dynamic Process	Stainton et al [39], 2019
**Resilience in health care systems (n=9)**		
	Exploring the Nature of Adaptive Capacity for Resilience in Healthcare Across Different Healthcare Contexts; a Metasynthesis of Narratives	Lyng et al [40], 2022
	Resilience in Organization-Related Research: An Integrative Conceptual Review Across Disciplines and Levels of Analysis	Raetze et al [41], 2022
	Shifting Focus from Burnout and Wellness toward Individual and Organizational Resilience	Vercio et al [42], 2021
	Health System Resilience: a Literature Review of Empirical Research	Biddle et al [43], 2020
	Measuring the Resilience of Health Systems in Low- and Middle-Income Countries: a Focus on Community Resilience	Bhandari and Alonge [44], 2020
	Maintaining capacity in the health care system during the COVID‐19 pandemic by reinforcing clinicians’ resilience and supporting second victims	Strametz et al [45], 2020
	COVID-19: Peer Support and Crisis Communication Strategies to Promote Institutional Resilience	Wu et al [46], 2020
	Strengthening Health Systems Resilience: Key Concepts and Strategies	Thomas et al [47], 2020
	Conceptual Analysis of Health Systems Resilience: A Scoping Review	Turenne et al [48], 2019
**Existing intervention programs to enhance resilience (n=6)**		
	Psychosocial Interventions for Building Resilience of Informal Carers of People Living with Stroke: a Systematic Review	Qureshi et al [49], 2023
	Building Personal Resilience following an online Resilience Training Program for BScN Students	Stoliker et al [50], 2022
	A Resilience-Building App to Support the Mental Health of Health Care Workers in the COVID-19 Era: Design Process, Distribution, and Evaluation	Golden et al [51], 2021
	The Effectiveness of Charge Nurse Training on Leadership Style and Resiliency	Spiva et al [52], 2020
	Comprehensive Meta-analysis of Resilience Interventions	Liu et al [53], 2020
	Interventions to Improve Resilience in Physicians Who have Completed Training: A Systematic Review	Venegas et al [54], 2019
**Useful tools (n=9)**		
	PERMA+4: A Framework for Work-Related Wellbeing, Performance and Positive Organizational Psychology 2.0	Donaldson et al [19], 2022
	Effectiveness of a One Day Self-Compassion Training for Pediatric Nurses’ Resilience	Franco and Christie [55], 2021
	The Positive Functioning at Work Scale: Psychometric Assessment, Validation, and Measurement Invariance	Donaldson and Donaldson [56], 2021
	The PERMA-Profiler: A Brief Multidimensional Measure of Flourishing	Butler and Kern [57], 2016
	The Behavior Change Technique Taxonomy (v1) of 93 Hierarchically Clustered Techniques: Building an International Consensus for the Reporting of Behavior Change Interventions	Michie et al [22], 2013
	G.R.A.C.E. for Nurses: Cultivating Compassion in Nurse/Patient Interactions	Halifax [20], 2014
	RAG—Resilience Analysis Grid	Hollnagel et al [21], 2011
	US Marine Corps and Navy Combat and Operational Stress Continuum Model: A Tool for Leaders	Nash [18], 2016
	Caring for Our Own: Deploying a Systemwide Second Victim Rapid Response Team	Scott et al [58], 2010

The working team outlined a presentation structure for eResiliencia and chose the most suitable appearance from several options. With the information gathered and the ideas from the working group itself, the first draft of the content was structured in the beta version of the website that hosts eResiliencia [59].

**Figure 3:**
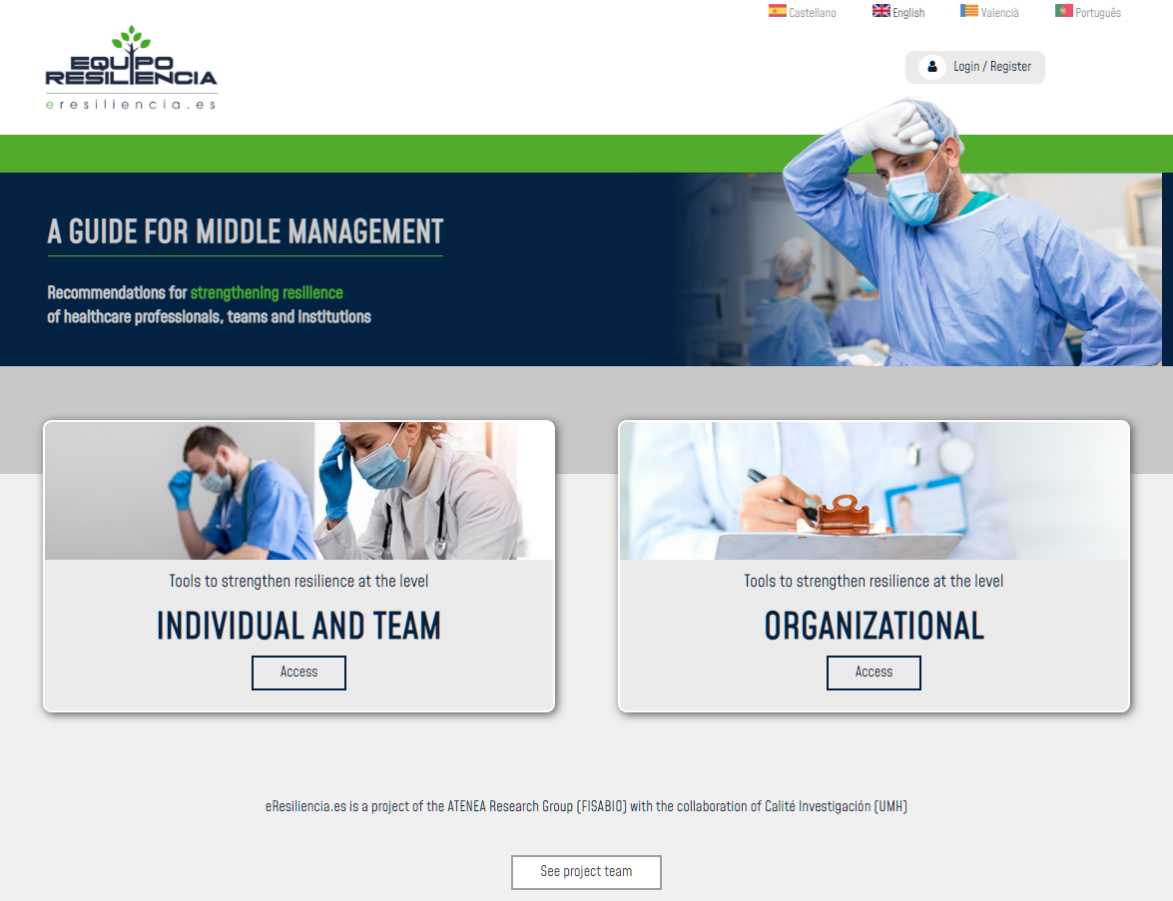
The home screen of the eResiliencia web-based platform.

**Figure 4:**
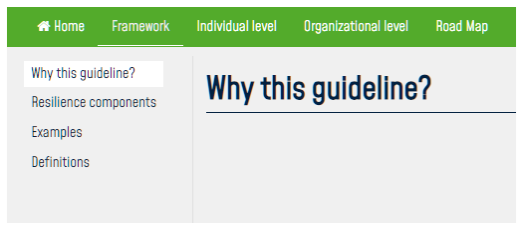
Navigation panel of the eResiliencia web-based platform.

The content was divided into 2 main blocks (Figure 3), one aimed at building resilience at the individual and team levels, and another at the organizational level, preceded by a conceptual framework. Platform navigation was conducted through both the menu bar and the side panel (Figure 4). Within each block, there were subdivisions as shown in Table 2. In the tab dedicated to the conceptual framework, an explanation of the course context, an introduction to resilience, several examples of situations where it is crucial, and a section with definitions of the most relevant concepts of the course were provided. The first block, tools to strengthen resilience at the individual and team levels, offered the necessary strategies to enhance the resilience of team members at an individual level, placing particular emphasis on the importance of workplace well-being. Finally, the block on tools to reinforce resilience at the organizational level provided strategies to assess and achieve resilient institutions in the face of crises and unexpected events. In addition, a road map was included at the end of the course to aid in selecting the most useful strategies based on the objectives.

**Table 2:** Structure of the eResiliencia web-based platform.

Structure	Items
Framework	Why this guideline? Resilience components Examples Definitions
Individual level	Stress Continuum Model Enhancing resilience at the individual level G.R.A.C.E. model PERMA+4 Well-Being model for occupational wellness Behavior change technique for building work-related well-being
Organizational level	Enhancing resilience at the organizational level Resilience Analysis Grid

In total, it comprised 17 videos, 11 figures and images, 4 PDF files, and references for further information expansion, among which downloadable materials or video, graph, and text links were also available. Upon accessing their personal account, users were provided with a section containing pertinent information regarding the platform’s functionality, along with a video elucidating navigation and content. In addition, users had access to a support resource and the option to change their password.

### Phase 2: Validation by Experts

Of the 20 experts, 13 (65%) assessed the tool and provided feedback. They rated the clarity of the content at an average of 4.5 (SD 0.7) and the utility and applicability at an average of 4.7 (SD 0.5), out of 5 in both cases. Regarding whether the materials facilitated learning strategies to foster the resilience of professionals or teams under their purview, 62% (8/13) of the experts indicated they *completely agree*, 31% (4/13) of the experts indicated they *agree*, and 8% (1/13) of the experts indicated they *completely disagree*. Regarding their likelihood to recommend the tool to other colleagues, 92% (12/13) of the experts responded *definitely yes*, and 8% (1/13) of the experts responded *probably yes*. The same results were obtained for their intention to apply learned tools or strategies. The level of agreement among experts for the combined set of the 5 evaluative variables of eResiliencia was 0.79 (95% CI 0.65-0.92).

Overall satisfaction was rated with an average of 9.3 (SD 0.6) out of 10 points. Some of the strengths highlighted were the chosen content and the ease of navigation and presentation of the web-based platform. In contrast, areas for improvement included making the videos more engaging and the possibility of incorporating practical exercises or a forum for participant interaction. As a suggestion, an introductory video of the content and the functionality of the web-based platform was included (Multimedia Appendix 2) [60]. The full list of comments made by experts is presented in Multimedia Appendix 3.

### Phase 3: Pilot Study

Of the 458 professionals invited to participate, 362 (79%) registered on the platform. The preintervention and postintervention questionnaires of the pilot study were completed by 218 (60.2%) of these 362 health care professionals exercising leadership roles who began the intervention. Moreover, 146 (40.3%) health care professionals responded to the experience evaluation questionnaire.

Most of them were physicians (90/218, 41.3%) and nurses (59/218, 27.1%), with a mean experience of 10.3 (SD 8.8) years. Their primary work setting was primary care (113/218, 51.8%), hospital (79/218, 36.2%), quality area (9/218, 4.1%), or other management positions (7/218, 3.2%). The profile of those who did not complete the entire educational intervention was similar to those who did, with 42.4% (61/144 being physicians, 24.3% (35/144) being nurses, and 45.1% (65/144) working in primary care. Those who did not complete the intervention had slightly average experience, with 11.4 years compared with 10 years.

The scores obtained in the preintervention questionnaire averaged 42.0 (SD 16.6) of 100, while for the postintervention questionnaire, they averaged 74.7 (SD 22.7), with significant differences between them (*P*<.001; *Z*=–11.728). The increase in the percentage of correct answers for each question is represented in Table 3.

**Table 3:** Increase in percentage of correct answers for each question (n=218).

Questions^a^	Correct responses on the preintervention questionnaire, n (%)	Correct responses on the postintervention questionnaire, n (%)	Increase of correct answers (%)	McNemar test	*P* value
1	148 (67.9)	192 (88.1)	20.2	25.681	<.001
2	73 (33.5)	148 (67.9)	34.4	53.165	<.001
3	102 (46.8)	155 (71.1)	24.3	27.876	<.001
4	32 (14.7)	134 (61.5)	46.8	87.940	<.001
5	103 (47.2)	165 (75.7)	28.4	37.969	<.001
6	66 (30.3)	176 (80.7)	50.5	99.008	<.001
7	92 (42.2)	162 (74.3)	32.1	52.900	<.001
8	110 (50.5)	180 (82.6)	32.1	48.582	<.001
9	94 (43.1)	153 (70.2)	27.1	30.862	<.001
10	82 (37.6)	159 (72.9)	35.3	55.010	<.001
11	139 (63.8)	189 (86.7)	22.9	29.280	<.001
12	117 (53.7)	169 (77.5)	23.8	28.900	<.001
13	44 (20.2)	165 (75.7)	55.5	111.628	<.001
14	110 (50.5)	181 (83)	32.5	49.495	<.001
15	29 (13.3)	111 (50.9)	37.6	64.324	<.001
16	86 (39.4)	145 (66.5)	27.1	33.307	<.001
17	138 (63.3)	194 (89)	25.7	34.375	<.001
18	114 (52.3)	154 (70.6)	18.3	20.554	<.001
19	84 (38.5)	162 (74.3)	35.8	55.934	<.001
20	68 (31.2)	163 (74.8)	43.6	73.025	<.001

^a^Because this is active training, the questions have been masked to avoid spoilers. They are available upon reasonable request.

Moreover, 58.3% (127/218) of the health care professionals passed the postintervention questionnaire without requiring any retries. However, 14.2% (31/218) of the health care professionals needed 1 retry, 8.3% (18/218) needed 2 retries, 6% (13/218) needed 3 retries, and 3.7% (8/218) needed between 4 and 7 retries. Furthermore, 9.6% (21/218) of the health care professionals exhausted all 10 retry attempts without achieving the required score.

Regarding the perception of the 146 people who completed the evaluation questionnaire, the clarity of the content received an average score of 4.7 (SD 0.6) on a 1 to 5 scale, while the utility or applicability scored 4.8 (SD 0.5). The results of their opinion are depicted in Table 4.

Global satisfaction was rated 9.4 (SD 1.1) out of 10 points on average. Among the strengths underscored were the comprehensiveness of the content and the interesting techniques explained. Conversely, identified improvement areas consisted of incorporating more illustrative examples and providing downloadable materials.

**Table 4:** Results of the nonnumerical scale questions from the assessment questionnaire (n=146).

Question	Completely disagree, n (%)	Disagree, n (%)	Neither agree nor disagree, n (%)	Agree, n (%)	Completely agree, n (%)	Definitely not, n (%)	Probably not, n (%)	Not sure, n (%)	Probably yes, n (%)	Definitely yes, n (%)
The course content has enabled me to learn strategies for reinforcing the resilience of the professionals or teams under my supervision.	7 (4.8)	1 (0.7)	6 (4.1)	54 (37)	78 (53.4)	—^a^	—	—	—	—
I will recommend the course to other colleagues.	—	—	—	—	—	0 (0)	0 (0)	6 (4.1)	22 (15.1)	118 (80.8)
I will apply some of the tools or strategies learned to strengthen individual, team, or organizational resilience in my work environment.	—	—	—	—	—	0 (0)	2 (1.4)	2 (1.4)	28 (19.2)	114 (78.1)

^a^Not applicable.

### Qualitative Analysis of Open-Ended Comments

#### Strengths

The most repeated topics among the strengths were the learning of new techniques or specific models (34/146, 23.3%), the web-based methodology that allowed access at any time (14/146, 9.6%), the organizational approach (10/146, 6.8%), the clarity of the content (9/146, 6.1%), stress management (6/146, 4.1%), and the examples provided (4/146, 2.7%).

#### Opportunities for Improvement

In terms of aspects to be improved, the incorporation of more examples (14/146, 9.6%) and the expansion of the content (14/146, 9.6%) were generally highlighted. In addition, improving the quality of the videos (12/146, 8.2%), including the possibility of interacting with other students in a more practical way (9/146, 6.2%), enabling downloadable content (9/146, 6.2%), reducing the length of the questionnaires (6/146, 4.1%), including web-based tutorials for resolving doubts with the teachers (5/146, 3.4%), and adding a progress bar to visualize the course as a whole (4/146, 2.7%) were emphasized.

## Discussion

### Principal Findings

Our developed web-based approach operates as a comprehensive tool designed primarily for middle managers, aiming to foster resilience at both individual and organizational levels within the staff at their charge. By using multimedia resources, this approach has been shown to enhance the knowledge about resilience of health care professionals across diverse fields and backgrounds.

eResiliencia was highly rated by the panel of experts regarding clarity and usefulness, indicating that the tool is perceived as comprehensible and relevant for application in professional contexts. High ratings were also obtained for the willingness to recommend the tool to colleagues and the intention to apply the learned strategies, demonstrating strong acceptance and confidence in the resource. For most (12/13, 92%) participants, the materials were potentially effective in strengthening team resilience, except for 1 (8%) expert who expressed complete disagreement. This highlights the need to consider diverse perspectives and possible improvements in adapting the content to various contexts. Nonetheless, overall satisfaction was high, with more than 9 points out of 10.

### Implementing eResiliencia in Real-World Settings

The eResiliencia platform features a user-friendly interface, and in most cases, managerial staff possesses the necessary skills to use such digital tools effectively. However, some systemic limitations may impact its implementation. First, the increasing measures to prevent cyberattacks and data ransom incidents in health care institutions have led to stricter internet access controls, even in areas where connectivity was previously unrestricted. This decision may hinder access to eResiliencia within some organizations. In addition, in certain health care networks, managers lack the resources to access the platform outside their offices. Incorporating offline functionality could address these limitations and facilitate broader access to the platform.

Second, resistance from health care organizations, often rooted in established workflows or skepticism about the benefits of new interventions, represents a traditional barrier. In this case, the integration of eResiliencia was facilitated by embedding it within a national patient safety and quality training program aimed at launching a global institutional strengthening plan. A similar strategy could help overcome organizational resistance in other settings.

Third, ensuring flexibility and accessibility is essential to meet the diverse needs of users. The use of asynchronous learning options was key in this instance, allowing participants to decide when and how to engage with the platform. This flexibility significantly increases accessibility and is particularly relevant in environments with high workloads. However, allocating dedicated time during work hours could further enhance participation rates by reducing competing demands on participants’ schedules.

### Comparison With Prior Studies

It is known that health care professionals face stressful situations in their professional work. The frequency of burnout or the existence of defensive practices is also not uncommon. It is no surprise that the role of team leaders influences the team’s ability to respond and recover after experiencing adverse situations [61]. The COVID-19 pandemic brought to the attention of managers, executives, and society in general concerns about the resilience capacity of these collectives, both at an individual and collective level, to face what has undoubtedly been the greatest global challenge for health care professionals. This study originates from that period and delves into both aspects, offering an easily accessible training format for those responsible for clinical teams. In this regard, it is based on the observation that most programs designed to increase resilience were developed to operate at the individual level and that it was necessary to create interventions focused on the work group [30].

eResiliencia incorporates agreed-upon resilience attributes [37] and skills that leaders must possess to strengthen their teams’ resilience, which include self-efficacy, self-control, the ability to provide support and help, and learning from difficulties [62].

Uncertainty, resource shortages, constant protocol variations, the risk of infection and transmission, and critical decision-making with ethical implications, among other factors, were prevalent during the COVID-19 pandemic and contributed to fear, anxiety, stress, moral injury, or compassion fatigue among health care professionals [63,64]. These situations have highlighted the need to reconsider the role of middle management, as they must address the needs of their teams to face challenges. Therefore, their resilience is a crucial competence required for their work.

The scores obtained in this study by the professionals who underwent the training show that after the use of the tool, the results were better both overall and for each individual question. However, the preintervention questionnaire also revealed that participants’ previous knowledge was relatively low in many areas, with correct response rates ranging from 13.3% (question 15; behavior change technique) to 67.9% (question 1; definition of resilience). This underscores the necessity and effectiveness of the educational intervention implemented. The greatest increases were in questions with initially low correct response rates (question 4, question 6, and question 13), suggesting the acquisition of new concepts. In contrast, questions such as question 1 and question 18, which initially had a higher percentage of correct responses, showed a less pronounced increase compared to those with lower initial performance. The increase in scores on the final questionnaire compared to the initial questionnaire is consistent with the findings of other studies, which show that both in-person [52,65-67] and on the web [50,68] interventions increase resilience capacity. In addition, it has been observed that they also protect the mental health of professionals [69].

Solutions to assist professionals were implemented in different countries and were similar, including the availability of psychological services, support hotlines, and websites with resource materials [70]. Focusing on tools available for Spanish speakers, an example is the digital platform and mobile app, Be+Against COVID, which was used in Argentina, Brazil, Colombia, Chile, Ecuador, and Spain [71].

Although middle managers have usually received training in occupational risk prevention and conflict management, many times there is a lack of specific and detailed training in fundamental aspects of team management and resilience. Traditionally, team management has focused on organizational and clinical aspects to ensure that patients’ needs are adequately met. Nowadays, middle managers are expected to efficiently manage the resources of the professional teams they lead, particularly human talent, to adequately respond to health challenges.

### Implications of Findings

In the postpandemic era, there is a growing consensus that among the functions of executives and middle managers (such as the organization of responses to care demands, task allocation, conflict management within teams, and the strengthening of teams to meet care demands effectively), there should be an emphasis on maintaining and strengthening the resilience capacity of their teams of professionals, because their ability to cope with highly stressful situations determines the quality of care provided and the outcomes for patients. This eResiliencia program follows this trend and takes a proactive approach by offering a framework for executives and middle managers to use some or all the resources to achieve this objective and prepare their team for everyday challenges and future crises.

Although most (197/218, 90.4%) of the participants were able to pass the training without any difficulties, some (21/218, 9.6%) individuals exhausted all reattempts without achieving the minimum required score. While these data suggest that the material and teaching methods are effective for most, it would be beneficial to delve into the reasons behind the difficulties faced by this small percentage to address and mitigate the problem.

Resilience training is presented as a crucial element in the professional development of middle managers in the health care sector. Promoting and reinforcing resilience is not only essential for the individual well-being of professionals but also strengthens the cohesion and effectiveness of the team as a whole. However, for this to be feasible, it is essential that clinical management evolves and provides effective tools to middle managers, facilitating resource management, including resilience training.

In this era, there is a growing consensus that the existing “find and fix” solutions should be replaced by those that are more forward-thinking [15]. Moreover, the organizational culture is critical to ensuring patients receive adequate care and workers’ resilience when things go wrong [72]. Health care organizations are evolving toward the principles of Safety II framework here [73], which involves recognizing the challenge of complexity and uncertainty in clinical practice to anticipate errors. Promoting the resilience of workers at both individual and team levels contributes to realizing the implementation of Safety II [74].

Fragmentation in the assignment of professionals poses a significant challenge to teamwork. Thus, cohesion among middle medical and nursing managers is essential to overcoming the barriers created by this fragmentation and fostering a more collaborative and efficient work environment. The training of middle managers in the health care organization must be a top-down strategy, driven by leadership. Health care executives must communicate these priorities to middle managers and provide the necessary support for implementation. Only through strong and consistent leadership from the upper echelons can we ensure that middle managers are equipped with the competencies required to effectively manage their teams and promote a resilient work environment.

The response rate indicates that 4 (40%) of the 10 professionals who registered on the platform were eventually not interested in the training on how to foster resilience in their teams and collaborators. This could be because they already possess the personal resources to tackle the task or because eResiliencia did not meet their expectations or failed to motivate them sufficiently. This aspect could be addressed, for example, with a more practical approach through group dynamics and cooperative tasks to generate greater interest and engagement, so participants can see a more direct application of the training tools.

### Strengths

This study addresses the 5 key questions of a resilience study in the health care sector [75]. It proposes an alternative approach to individual-focused training by emphasizing the role of clinical team leaders and how to strengthen the resilience of their teams and team members.

### Limitations

Although international experts share a common cultural background, they represent a group of Spanish-speaking countries. The generalization of content to other countries should not be done directly. Participants in the pilot study phase worked in various public health networks in Ecuador and took part in this experience as part of a quality and patient safety management course, which emphasized the need to move away from a blame culture. This aspect may have influenced their engagement with the eResiliencia content. Participants had a set amount of time to complete the training on the eResiliencia web-based platform. In a different self-directed learning context without this limitation, trainee behavior might be different. Because this is a study without a control group, the changes observed in participants’ knowledge may not be exclusively attributable to eResiliencia. Finally, it should be noted that although the participants were enrolled in training organized by the Ministry of Public Health of Ecuador, it was emphasized that they could freely choose whether to complete the eResiliencia training and respond to the satisfaction survey. Answering the questionnaire did not provide any advantage and the responses were not disclosed, ensuring anonymity at all times, which was clearly communicated to the participants. This approach was taken to avoid a reporting bias.

### Cultural Factors Influencing Resilience-Building Capacity

Ecuador’s health care system is characterized by hierarchical structures and centralized decision-making, typical of high-power distance cultures. eResiliencia capitalizes on the influential role of middle managers, who act as intermediaries between upper management and frontline workers. By equipping middle managers with resilience-building tools, the intervention empowers them to cascade these strategies throughout their teams, reinforcing resilience at all levels of the organization. The networks engaged in this study operate with limited resources, requiring flexibility and improvisation to address challenges. eResiliencia provides structured yet adaptable strategies, offering tools for middle managers to tailor resilience-building efforts according to the specific needs and constraints of their teams. Although Ecuador faces challenges in long-term health care planning, eResiliencia introduces structured frameworks that emphasize proactive resilience building. These frameworks align with preventive measures to prepare health care professionals for crises, fostering a shift from reactive to proactive management.

Many of these aspects are common to countries in this region. However, the outcomes of eResiliencia in Asia, Europe, or North America are likely to differ significantly due to the cultural factors influencing resilience-building capacity. Cultural dimensions such as collectivism, power distance, and approaches to uncertainty, among others, play a crucial role in shaping how resilience strategies are developed and applied across different contexts.

### Future Research

It is necessary to delve into a common definition of what is meant by resilience [11,75] and, by extension, the role of managers in the resilience of their team members. A critical aspect for future development involves the incorporation of mechanisms to monitor the utility and long-term impact of the knowledge acquired through the web-based tool, particularly considering the less optimistic data provided by some studies on nursing students [76]. In later phases, this intervention will be complemented by the development of improvement and action plans based on the application of the eResiliencia tools by health care teams and middle management interested in their centers. This will allow a more effective monitoring of the tool, its usefulness, and applicability. These approaches could also be extended to companies that provide home care, to train those who coordinate the work of informal caregivers in the home [77], in light of the growing caregiving economy. Finally, as these are preliminary data, this educational intervention should be tested in the context of other organizational models and diverse cultural settings to validate its effectiveness, acceptability, and utility.

### Conclusions

The proposal to evaluate each professional, identify signs of stress, take appropriate measures, and manage team cohesion represents a training initiative that could be implemented clearly and explicitly. However, it is essential to first analyze the organizational context where the intervention will be applied and identify potential adjustments to improve its acceptability based on national and local organizational factors. This tailored approach ensures that the intervention aligns with the specific needs and characteristics of each setting, enhancing its effectiveness and relevance. This training would enable middle managers not only to manage conflicts but also to prevent them and foster a healthy and productive work environment. The developed tool proved to be well received by both the experts and the health care professionals who participated in this pilot phase.

## Appendix

Multimedia Appendix 1 Topics of the preintervention and postintervention questionnaire questions.

Multimedia Appendix 2 eResiliencia introductory video.

Multimedia Appendix 3 List of comments received from the experts during the validation phase.

## Data Availability

The datasets generated analyzed during this study are available from the corresponding author on reasonable request. Topics addressed in each question are displayed in Multimedia Appendix 1.
