# Supplementary material for: Development of a Web-Based Intervention for Middle Managers to Enhance Resilience at the Individual, Team, and Organizational Levels in Health Care Systems: Multiphase Study
Source: JMIR Hum Factors. 2025 Feb 5;12:e67263. doi: 10.2196/67263 (PMC11840388; doi:10.2196/67263)
Supplement: Multimedia Appendix 1 [file humanfactors_v12i1e67263_app1.doc]

## Topics of the pre- and post-questionnaire questions.

**Q1:** Definition of resilience.

**Q2:** Impact of stoicism on healthcare professionals.

**Q3:** Quadruple Aim Model.

**Q4:** Agents for foster resilience in healthcare settings.

**Q5:** Personality traits associated with resilience.

**Q6:** Origin of the Stress Continuum Model.

**Q7:** Zones in the Stress Continuum Model.

**Q8:** Zones in the Stress Continuum Model.

**Q9:** Psychological health of healthcare professionals.

**Q10:** Susan Scott’s Three-Level Support Model.

**Q11:** Psychological first aid principles.

**Q12:** Resilient attitudes in the collective context.

**Q13:** Components of the PERMA+4 Model.

**Q14:** Components of the PERMA+4 Model.

**Q15:** Behavior change technique.

**Q16:** Work-related well-being.

**Q17:** G.R.A.C.E. Model.

**Q18:** Features to enhance resilience at the organizational level.

**Q19:** Capabilities for resilient system performance.

**Q20:** Strategies to reinforce resilience at the macro level.
