# Supplementary material for: Development of a Web-Based Intervention for Middle Managers to Enhance Resilience at the Individual, Team, and Organizational Levels in Health Care Systems: Multiphase Study
Source: JMIR Hum Factors. 2025 Feb 5;12:e67263. doi: 10.2196/67263 (PMC11840388; doi:10.2196/67263)
Supplement: Multimedia Appendix 3 [file humanfactors_v12i1e67263_app3.doc]

## List of comments received from the experts during the validation phase.

|  | **Strengths** | **Aspects to be improved** |
| --- | --- | --- |
| **E01** | - Clarity of examples and recommendations to be implemented. | - None. |
| **E02** | - I find everything well developed. - I find the web-based platform appropriate and well-structured. | - In the roadmap, a distinction is made between strengthening the resilience of a team member and strengthening the resilience of the organization, but there is no mention of strengthening personal or individual resilience as indicated. If strengthening the resilience of a team member is considered the individual level, perhaps it would be clearer and more consistent with the different sections to refer to it as strengthening individual resilience. - Perhaps what’s missing is measurement, that is, an evaluation of whether we have achieved the objective of improving individual and group resilience. I'm not sure if there is a "measurement tool" for this. - Since it may be aimed at different groups, a relevant question might be: Does this training apply to any professional group or only to medical and nursing staff? I’m not sure if it is suitable for all professional groups. It would be good to test this. - Increasing resilience through training, for example, has a preventive or health promotion objective for workers. It would be interesting to emphasize this and not focus solely on the issue of safety incidents. |
| **E06** | - Ease of navigation. - Conciseness of the presentations. - The objectives set are met. | - The videos, I would incorporate videos or other elements that allow them to be more dynamic. |
| **E07** | - The content is easy to understand and apply. - The material presented and the bibliographic resources. | - I believe that the training contributes to and addresses very well the strengthening of resilience in individual and organizational clinical practice. |
| **E08** | - The web-based platform brings an overview of stress mechanisms and how to develop middle management to recognize and support them. - I liked it very much because I see that the (…) reality would benefit from this universe of information. | - The sound of the videos: they vary in intensity. - The remaining link to take the personal resilience test does not open. - It is not clear if there is any instrument that can measure or assess the questions presented in RAG. |
| **E09** | - The contents are concise and clear, very specific to the realities of the organizations. | - I would incorporate didactic tools for assessing comprehension. |
| **E10** | - Despite my previous experience, I discovered things that opened my eyes and expanded possibilities for managing my team. So, from the outset, I find it very useful. - I really like the recommendations for enhancing both individual and collective resilience. - I believe the planning matrix is a very useful tool for middle management. | - The presentation and development of the PERMA + 4 model seemed overly extensive. Perhaps simplifying its presentation and providing additional material for those who want to dive deeper would be beneficial. - I wasn’t clear on the role of the Donaldson scale until the end when I saw the roadmap. What I mean is that I might place the roadmap at the beginning to better understand the development of the training. - The GRACE model is excellent, but it felt a bit out of context when introduced at the end of the chapter. - I don’t fully see the need for a separate section on organizational resilience, as it introduces elements beyond the decision-making scope of middle managers. - I feel there is a lack of a passionate presentation of the role of middle managers. The presentations are somewhat plain in their explanations, which is a common challenge in online training. Seeing the faces of those who prepared the training and conveying the strong conviction about the importance and responsibility we middle managers have in team cohesion, while providing basic and useful tools for managing the resilience of professionals, seems important to me. |
| **E13** | - I think it’s a fantastic project. The topic of resilience has really piqued my interest. In our department, we had a session on it prepared by the (…) psychologists who work with us. Group cohesion seems fundamental to me. - The videos are short and user-friendly, which helps avoid information overload. - The web-based platform seems very complete in terms of content and highly necessary. | - The video on the Stress Continuum Model is the only one that feels less fluid. The oral information in the initial chart doesn’t match exactly with the written content, and the written content isn’t as easy to follow and understand—it’s easy to get lost in the details. - Regarding the roadmap, I’m not sure if it's intended to be complemented with the "how" of PERMA +4 and the RAG, in relation to the behavior change technique and the charts highlighting the critical points that are referenced. |
| **E14** | - Practical examples and action guidelines. | - Only the volume of the voiceover in some videos. |
| **E15** | - It’s great and very interesting to include the *Learn More* section in all areas. Congratulations. - The downloadable materials are intuitive. They’re useful and encourage action and application of the training. - I found it very interesting overall, and if you agree, I’d like to learn more about its direct applicability. | - In the initial phase, when seeing the opening questions, they can be a bit frustrating as they refer to concepts that are difficult to answer if you haven’t taken a prior specific training. However, there are other conceptual questions that are very interesting. Perhaps the focus shouldn’t be on asking whether someone knows a model or paradigm but rather on conceptual questions, followed by an explanation of what the model will contribute during the training. - At the start of the program, I would indicate an estimated time for completion. Perhaps two or three hours, plus the practical development. I also think a more comprehensive introduction to the activity would help, describing the common thread that ties together the different sections, linking more clearly the *Introduction* and/or *Objectives* with the *Roadmap* section. - The voiceover in *Why this guideline*, which is used in other videos, feels a bit too plain. In some cases, it simply reads the content on the slides word for word. This sometimes makes it tempting to skip through the presentation… - In section 1.1 *Why this guideline?*, compassion fatigue is mentioned. This is a concept I believe is not widely known. A definition could be added. - In section 1.2, perhaps cumulative transitions in the slides could reinforce the content being presented, though I’m not sure. - In section 1.3, the examples are analyzed through three elements (comprehension, management, and meaning), but it wasn’t clear to me why these three elements are used. - In the section *Functions of Middle Managers to Promote Psychological Health at Work*, I’m unsure whether the prevention service technicians are in level two or three of the Scott model. If we’re only discussing adverse events (within the safety framework), then yes. But when it comes to dealing with stress issues, I’m not so sure they should be placed directly in the second level. This could lead to confusion. In the section on the 7 pillars, pillar 7 is labeled as competence, but it’s actually pillar 6. A small error in the speech. - The *Functions of Middle Managers* section is the longest. In the section on *Enhancing resilience at the individual level*, it can be directed to any worker, not just managers. Some kind of material could be provided that managers can share or distribute among their workers. - To me, there is a gap between the *Stress Continuum Model* and *Enhancing resilience at the individual level* with the PERMA+4 model (along with behavior change technique). The PERMA section presents a broad, complex theoretical model that, in my view, should be adopted by the entire organization, not just the manager. I’m a bit concerned that too much responsibility and expectations are being placed on middle managers. It might be worth softening this or distributing the possibility of introducing this change across organizational, individual, and managerial levels. - Related to the previous point, in the section on *Behavior change technique for building work-related wellbeing*, it might also be helpful to guide managers to focus on areas where they can or should develop more, rather than trying to address everything. - In the GRACE section, it could be more clearly connected to the rest of the training content. Its focus shifts somewhat. Emphasize that it focuses on burnout, a specific tool that can be a source of resilience, but also for improving health and wellbeing directly. Like in the *Enhancing resilience at the individual level* section, this content can be aimed at any worker, not just managers. Some material could be provided for managers to share with their workers. - In the section on the analysis table, it might be interesting to encourage interaction among participants taking the training. If they are from the same organization, propose that they interact, or suggest having a repository where, without naming the organization (unless desired), notable examples or best practices are shared. |
| **E16** | - I really like the approach, and the design complements it well. | - I would only organize it in a format more like an itinerary, where progress can be tracked. - I understand that the questions in the questionnaire have their corresponding justification. |
| **E17** | - The contents are a strong point. | - Perhaps I would include a screen with the objective of the presentation and one at the end as a summary. - Image of the speaker, when they take a while with just the voice it becomes monotonous. - The presentation could be improved to attract more attention, less text and more image. |
| **E18** | - The initial questionnaire, which is repeated at the end, contains questions that can be easily answered in the modules. - The video content for both the individual and organizational levels is well structured and easy to understand. - The character vignettes with their messages in the first video are very helpful. Perhaps this could have been applied to all modules. - I think this training is very relevant right now, particularly for middle managers who are experiencing burnout and struggling to motivate their teams. | - The only thing is that all the questions are all of the same type. Perhaps some matching or sentence completion questions could have been included instead of asking for definitions. Additionally, some questions could be based on practical case studies that apply the tools being proposed. - It might also be useful to include examples of specific activities they can do with their teams. - It could be considered that the students could share some case of application of the techniques so that they could be useful to others. |
